# Prevalence of Painful Temporomandibular Disorders and Correlation to Lifestyle Factors among Adolescents in Norway

**DOI:** 10.1155/2017/2164825

**Published:** 2017-05-30

**Authors:** Vegard Østensjø, Ketil Moen, Trond Storesund, Annika Rosén

**Affiliations:** ^1^Division of Oral and Maxillofacial Surgery, Department of Clinical Dentistry, University of Bergen, Bergen, Norway; ^2^Specialist Oral Health Centre for Western Norway, Stavanger, Norway

## Abstract

**Aim:**

To estimate the prevalence of painful temporomandibular disorders (TMD-P) among adolescents and to investigate correlations with health, environment, and lifestyle factors.

**Methods:**

For this cross-sectional case-control study, 562 patients were consecutively recruited at their yearly revision control from four dental clinics in Rogaland County, Norway. Patients completed a questionnaire on general health, socioeconomics, demographics, and lifestyle factors. Responses to two screening questions identified patients with TMD-P, who then underwent clinical examination to verify the TMD diagnosis. Pain intensity was assessed on a visual analogue scale. Patients without TMD-P constituted the control group and were not clinically examined.

**Results:**

7% experienced TMD-P. The female-to-male ratio is 3:1; median age is 17 years. Patients at urban clinics had higher prevalence compared with those at rural clinics. TMD-P patients had headache and severe menstrual pain compared to controls. They were more likely to live with divorced/single parents and less likely to have regular physical activity. Myalgia was present in 21 patients with TMD-P, arthralgia in nine, and myalgia and arthralgia in nine. Females had higher pain intensity than males.

**Conclusions:**

A low prevalence of TMD-P was shown but was comparable to other studies. Sex, health, lifestyle, and environment factors were associated with TMD-P.

## 1. Introduction

Temporomandibular dysfunction or disorder (TMD) includes disorders in the masticatory muscles, the temporomandibular joint (TMJ), or both [1]. Pain in the masticatory muscles or TMJ, impaired joint function, or a combination of factors is typical signs of TMD [2]. TMJ disorders are specific to the joint and encompass disc displacements and degenerative diseases [1]. Clicking or crepitation sounds from the TMJ as well as reduced mobility of the joints may occur [3]. TMD also includes myalgia, a nonspecific pain of muscular origin. Distinguishing between joint- and muscle-related disorders is important in the context of possible treatment. With correct diagnosis and treatment, pain resolves over time in most cases. Long-standing TMD without a correct diagnosis and treatment can sometimes give rise to chronic or persistent pain [1].

Historically, the etiology of TMD has been attributed to the dental occlusion, but this cause has become less clear-cut in recent decades [1, 4]. TMD is now considered to be multifactorial, with both somatic and psychological components. In particular, psychopathological conditions, such as anxiety and depression, seem to play a significant role in many patients with TMD [1, 5, 6].

At least one sign of TMD is found in 40% to 75% of adults in the United States [1]. In a Brazilian study of children and adolescents between 6 and 14 years of age, the prevalence of moderate or severe TMD signs, according to the Helkimo Index, was found to be 37.4% [7]. Severe TMD symptoms typically have a low prevalence [8]. In Sweden, some studies have shown a prevalence ranging from 4% to 7% of adolescents [9–11]. Women are 2 to 4 times more likely to develop TMD during their lifetime than men [12]. Among children and adolescents, girls have a significantly higher prevalence than boys [11], but the prevalence is lower than for adults. In Norway, only one epidemiological study on TMD among adolescents can be found in the literature [13]. The study used two protocols to estimate the prevalence of TMD (11.9%) and pain related to TMD (7.2%) in the same population [13].

In recent years, TMD has received increased attention in the Norwegian media. Reports emphasize the poor level of treatment provided in Norway as well as a lack of economic support from the country's welfare system for expensive treatment. As a consequence, many patients have sought treatment abroad [14].

According to a Norwegian national health survey, psychological problems like stress and anxiety among adolescents are greatly increasing in Norway [15]. This trend has been partly explained by self-applied pressure to be perfect in all aspects of life, including school, the social sphere, and physical appearance. This pressure has increased with the introduction of social media in daily life, such as Facebook, Snapchat, and Instagram, according to a Finnish study [16].

In Norway, dental treatment through the Public Dental Service (PDS) is offered free of charge to all children and adolescents up to 18 years and with a 75% reduced rate for young adults aged 19 to 20 years. Patients visit specific dental clinics based on their home address. These clinics are located throughout the county and serve the entire population. Private clinics may be located in the same areas (especially urban areas), but they serve the adult (>20 years) population, not children and adolescents. The dental care offered through the PDS is used by most children and adolescents. According to the 2014 annual report of the PDS, 98% to 99% of all children and adolescents in Rogaland County attended regularly scheduled appointments at recall intervals ranging from 6 to 24 months based on individually assessed risk and treatment needs. Consequently, the PDS and this age group are suitable for epidemiological studies because a representative selection of the population can be achieved.

Because TMD is multifactorial and includes several separate disorders, its diagnosis is a challenge. A widely used method is the Research Diagnostic Criteria for TMD (RDC/TMD) [2], and a revised protocol called Diagnostic Criteria for Temporomandibular Disorders (DC/TMD) has been published [2]. These protocols are comprehensive and may be difficult to implement on a daily basis in the clinical setting. A simplified method for screening TMD patients is the self-reported questionnaire pertaining to painful TMD (TMD-P), which consists of two questions [17]. In a validation study the questionnaire was found to have high sensitivity and specificity for diagnosing painful TMD, comparable to the RDC/TMD criteria [17]. More and larger studies on TMD-P in Norway are needed to assess treatment need in Norway.

The aims of this study were to estimate the prevalence of painful TMD according to the TMD-P criteria in an adolescent population, outline possible predisposing factors and possible factors of comorbidity, and describe some clinical key features of painful TMD.

## 2. Materials and Methods

This prospective cross-sectional clinical study was initiated by the University of Bergen and the PDS of Rogaland County, Norway.

### 2.1. Ethical Approval

This study was approved by the Regional Ethics Committee of Norway (REK-number 2013/171). All participants signed a written consent form. Participants age 16 or older signed on their own behalf, while parents signed on behalf of younger participants. Measures were taken to achieve full anonymity of all patients.

### 2.2. Study Sample

The patients in this study were randomly selected at the age of 13 to 19 years from 4 clinics in Rogaland County, Norway. We estimated that four clinics would be representative of the county, preferably two clinics in urban areas and two in rural areas to achieve a geographical spread. Eleven dentists working in four different clinics volunteered. One clinic was in a rural area and three were in urban areas. The study population represents 1.3% of the total population in the county. A total of 43,280 persons were between 13 and 19 years by December 2013 in Rogaland county. The average number for each year of age was 6183.

The number of clinics and dentists that performed the assessments accounts for 10% of all clinics and dentists in the county. The dental clinics were recruited via an invitation on the PDS website (website: http://www.tannhelserogaland.no/thr/information-in-english; the invitation: http://www.tannhelserogaland.no/tannhelse/aktuelt/invitasjon-til-aa-delta-i-forskningsprosjekt-om-tmd). Patients listed with the respective clinics were recruited continuously according to scheduled appointments (recall interval).

Patients with a scheduled recall appointment during the data collection period (August 2013 to June 2014) were included in the study. The clinics were given a number of patients to include, and they subsequently enrolled the patients based on recall lists. The clinics included the first 10 patients that were scheduled after the initiation of the data collection for both sexes and each age (i.e., 20 patients for each age, 10 of each sex). Based on previous studies with 4% to 7% cases, it was anticipated that a sample of 500 patients would result in approximately 30 cases. The argument for a minimum of 30 cases was that this number is generally considered sufficient to obtain adequate mean value estimates, which are close to normal distribution. Descriptive statistics were considered adequate for the purposes of this study.

To ensure that at least 30 cases were included and to achieve an even distribution between males and females, we included 40 persons of each sex per age in the study. A total of 560 patients were included in the study. Persons not able to answer questionnaires or adequately cooperate during a physical examination were excluded. This group was mainly mentally disabled persons (e.g., Down's syndrome). Patients, who actively sought dental treatment, including TMD treatment, were also excluded.

### 2.3. Screening and Questionnaire

**Figure 1:**
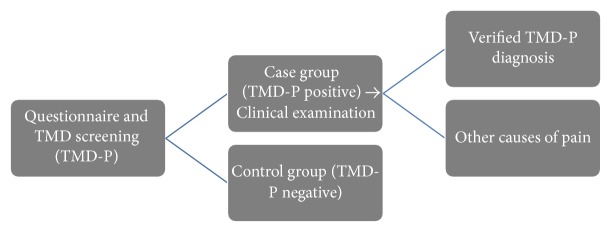
Flowchart showing the design of the study. Screening consisted of a questionnaire and two TMD-P screening questions. Patients with a positive TMD-P response underwent a clinical examination. Patients with a negative TMD-P response served as controls. The clinical examination revealed either actual TMD-P or other causes.

Patients were asked to complete a questionnaire before their routine oral health screening. Questions pertained to age, sex, family background, and general and special health information. The last two questions in the survey were the TMD-P screening questions. Patients who answered yes to one or both TMD-P questions underwent clinical examination of the TMJ joint and muscles in addition to their routine oral screening. If the pain was related to caries, infection, or other non-TMD cause, it would be uncovered during the routine oral screening. Patients answering no to both questions had a routine oral screening performed as usual. These patients constituted the control group (Figure 1).

### 2.4. The Design of the Questionnaires

#### 2.4.1. General Information, Education, Physical Activity, and Deskbound Time

The patients were asked to provide their age, sex, place of residence, and citizenship. Patients who were in high school were asked what kind of high school education they were receiving. The patients were also asked whether they were physically active, and those responding affirmatively were asked to indicate how many times per week. Participants were also asked how many hours per day they used a computer screen.

#### 2.4.2. Family Background

The patients were asked about their parents' background/citizenship. They had to mark their parents' marital status either as married/living together or as single. They were also asked for the number of children in the family, including stepsiblings.

#### 2.4.3. General Health Status

The questionnaire contained 13 dichotomous questions about specific diseases, including cardiovascular diseases, diabetes, epilepsy, cancer, psychiatric disorders, hemophilia, and disorders of the immune system, lung, stomach, liver, thyroid, and hormones. If asthma or allergies were present, the participants were asked to indicate triggers. They were also asked questions about smoking and dry snuff/tobacco use as well as comorbidities, such as headaches, rheumatoid arthritis, rheumatic fever, arthritis, hypermobility, muscular diseases, and ear, nose, and throat problems. Previous trauma to the jaws/face and neck injuries/whiplash were also addressed in the questionnaire, and other questions included orthodontic treatment and complications after dental treatment. Age of puberty was recorded. The girls were asked for their age at first menstruation, and the boys were asked for their age at which their voice broke.

The questionnaire ended with the TMD-P questions, which have been translated from Swedish to Norwegian [17]. These questions were as follows:

1. Do you have pain in your temples, face, temporomandibular joint, or jaws once a week or more?
2. Do you have pain when you open your mouth wide or chew once a week or more?

### 2.5. Design of the Clinical Examination

If answering yes to one or both TMD-P questions, a patient was asked to complete a second questionnaire and underwent a special clinical examination in addition to their regular oral screening. A resident in oral and maxillofacial surgery (OMS), trained by a specialist in OMS at the University of Bergen, gave standardized instruction to the 11 dentists who conducted the clinical examination. The two-day instruction included a thorough theoretical background about TMD and the components of a clinical examination according to the RCD/TMD criteria. On the first day, the theoretical background was presented and the dentists performed the assessment technique, including standardized pressure measurements, on each other. The second day included the clinical examination on actual patients in their respective clinics under the guidance of the OMS resident. Corrections were made to synchronize the dentists.

The additional questionnaire used for patients includes a visual analogue scale (VAS) to score the pain intensity (on a scale 0 to 100; 0 = no pain, 100 = worst, unbearable pain). Other questions centered on the frequency of symptoms and previous treatment of the condition. The clinical examination included assessment of jaw movements using a ruler (maximal opening between the incisal frontal teeth, laterotrusion, and protrusion in millimeters), registration of joint sounds (clicking or crepitations), and palpation for tenderness over the joints and masticatory muscles. Tenderness to palpation was recorded and graded as none, mild, moderate, or severe pain. A diagnosis of myalgia, arthralgia, or a combination of the two was given based on the findings. The examination was based on the RDC/TMD criteria for anatomical sites and digital pressure.

### 2.6. Statistical Methods

The data were coded and analyzed using STATA IC version 13 for Windows (College Station, TX). Differences between groups of categorical variables were analyzed by applying Pearson chi-square tests. For comparison of the TMD-P group to the control group for continuous variables, logistic regression analyses were applied. Student's *t*-tests were applied for continuous variables. *p* values less than 5% (*p* < 0.05) were considered statistically significant.

## 3. Results

### 3.1. Frequency

**Figure 2:**
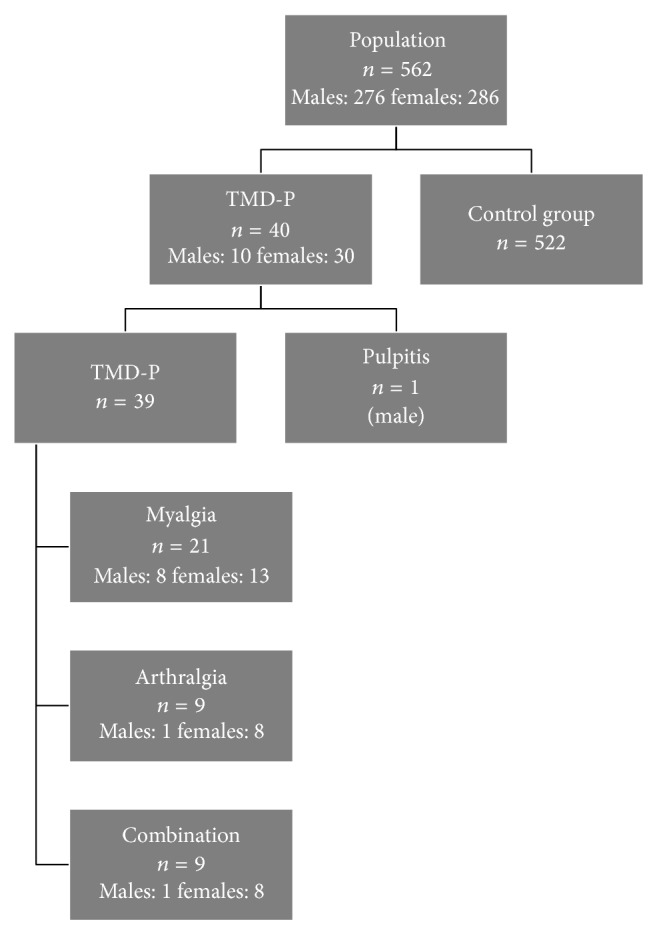
Flowchart showing the number of patients answering the TMD-P questions. The left column shows the case group with the distribution by sex. The right column shows the size of the control group.

A total of 562 patients, including 286 females (51%) and 276 males (49%), answered the two TMD-P screening questions. Forty patients affirmed TMD-P and composed the case group; the remaining 522 patients served as the control group. TMD-P prevalence in this cohort of 13- to 19-year-old Norwegians was 7% (Figure 2). Responses to the TMD-P screening questions were evenly distributed: 38% answered yes on question 1, 32% on question 2, and 30% of the patients answered yes on both questions.

### 3.2. Age, Sex, and Puberty

The female-to-male ratio in the case group was 3 : 1 as shown in Figure 2. This finding was statistically significant (*p* < 0.001).

**Figure 3:**
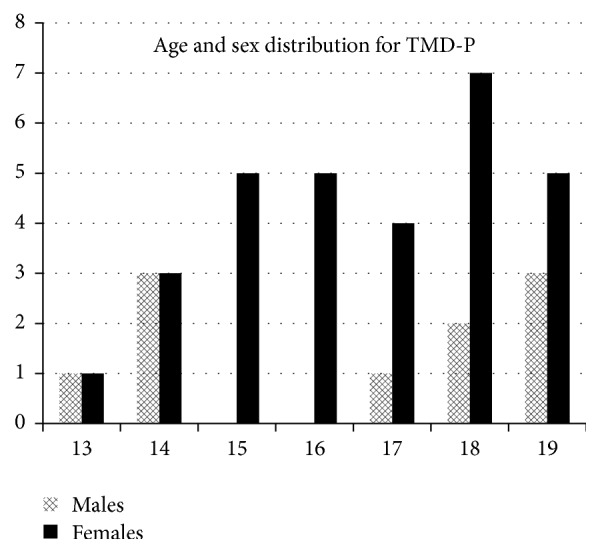
The age and sex distribution in the case group is presented. Females are highly overrepresented from the age of 15.

Mean age in the TMD-P positive group was 17 years (Figure 3). Bivariate logistic regression and chi-square tests did not show any significance for the role of age.

The mean age at menarche was 12.7 years, while the mean age of voice breaking was 13.2 years. The sex difference was statistically significant (*p* < 0.0001). Logistic regression corrected for sex showed a *p* value of 0.099 for increased risk of TMD-P after puberty (if not corrected for sex the *p* value was 0.028). Odds ratio (OR) for the age of puberty and TMD-P was 0.78, indicating a lower probability for TMD-P; the older a person was at puberty.

### 3.3. TMD-P in an Urban versus Rural Area

Three out of the four clinics participating in this study were in urban areas, while one was in a rural district. In proportion to the whole country this division was in accord with the Central Statistical Agency of Norway. The prevalence of TMD-P was 8.5% in the urban area clinics (*n* = 387) and 3.4% in the rural district (*n* = 175). This difference was statistically significant (*p* < 0.05).

### 3.4. Self-Reported Variables

#### 3.4.1. General Health

Some variables regarding general health, such as frequent headache and severe menstrual pain, were overrepresented in the case group in comparison to the control group (Table 1).

#### 3.4.2. Factors Related to Lifestyle and Family

Lifestyle factors and the family makeup can reflect how stressful daily life can be (Table 1). Living within a family with divorced or single parents was significant more likely for cases of TMD-P than for the control group. Regular physical activity was less frequent for TMD cases than for the controls. Spending more than 3 hours per day in front of a computer did not affect the prevalence of TMD-P.

#### 3.4.3. Trauma

None of the patients in the TMD-P group reported previous trauma to the face, but 12 patients in the control group had previously experienced facial trauma (Table 1). Half of the patients (6 of 12) who reported trauma to the face lived in the rural area.

#### 3.4.4. Previous Treatment

Only two patients had previously received treatment for TMD. In both cases, an occlusal splint was used.

### 3.5. Clinical Assessment

The range of motion was 35 to 60 mm for mouth opening, 1 to 17 mm for protrusion, and 5 to 17 mm for lateral movement of the mandible; 7.5% of the case patients could open their mouth less than 40 mm, 50% between 40 and 50 mm, and 42.5% more than 50 mm. Moderate or severe pain on palpation over the TMJs was reported in 45% of the case patients. Tenderness to palpation over at least one muscle of mastication was reported in 82.5%. Moderate or severe tenderness to palpation over the muscles of mastication ranged from 35% to 45%.

## 4. Discussion

This study is the second and by far the largest study on the prevalence of TMD-P among adolescents in a Norwegian county. The prevalence of TMD-P in Rogaland County was 7%. Another Norwegian study (*n* = 167) found a prevalence of 7.2% among adolescents in the city of Bergen based on the TMD-P questions, but the DC/TMD criteria for TMD indicated a prevalence of 11.9% [13]. The findings of the present study are in accordance with a Swedish study from 1999 that included 862 adolescents (12 to 18 years old), with a prevalence of TMD-P of 7% [11]. Another Swedish study published in 2005, using the TMD-P questions on more than 28,000 adolescents in the year 2000, found a prevalence of 4.2% [10]. The number of participants in the studies may explain these differences. The discrepancy may also be attributed to differences between societies in Norway and Sweden, despite these neighboring countries often being regarded as similar.

The 14 years between the studies has seen the introduction of smartphones and social media. Consequently, adolescents of today are more or less constantly online and introduced to new areas in which to perform. The Norwegian media frequently reports about social media, such as Facebook, Instagram, and Snapchat, and the negative impact they have on the psychological health of adolescents. A Canadian study from 2014 found that 23% of teenagers experienced cyberbullying the last 12 months. It also found that the teenagers had lower self-esteem and showed greater psychological distress than nonvictims [18]. A Norwegian national survey on adolescents reports an increase, especially among females, in anxiety and psychological distress from 2010 to 2013 [15]. This change in social interaction may explain some of the increased incidence of TMD-P from 2000 to 2014. Two other recent studies report a prevalence of TMD among adolescents ranging from 34% to 74% [19, 20]. These studies included both subjective (e.g., pain in movement) and objective (e.g., joint sounds) symptoms to estimate the prevalence, but they did not include the two TMD-P questions and therefore cannot be compared with this study. Such a high prevalence of TMD affects half the population, and it is worth discussing whether nonpainful TMD truly is TMD. In the oral and maxillofacial surgeon's environment this subject is often debated. According to DC/TMD criteria, clicking without pain is TMD; however, since this symptom is common and is seldom a problem for the patient, we do not think it should be classified as TMD. If so the classification will highlight a disease rather than normalize it. In addition, in the absence of subjective problems, the patient is automatically excluded from any type of treatment. The prevalence of asymptomatic disc displacement (pain-free clicking) in the normal population is 6% in 11-year-olds [21], 34% in 16- to 19-year-olds [22], and 31%–34% in adults [23, 24], and it indicates a physiological variant rather than a disease state [25]. Therefore, pain-free clicking is irrelevant in a clinical situation. Furthermore, there is no evidence that the clicking later develops into pathology or that preventive treatment would have any beneficial effect. In summary, these points motivate exclusion of pain-free clicking from clinical screening. However, using a screening instrument, such as TMD-P, is important for identifying potential candidates for TMD treatment.

As seen in this study, females were overrepresented among cases, with a ratio of 3 : 1 compared to males. Several other studies have found similar ratios, ranging from 2 : 1 up to 9 : 1 [10–13, 19, 20, 26, 27]. This study did not find such differences at ages 13 and 14, while other studies did not find differences before puberty [7, 28]. This study did not show the same result, but the OR for the age of puberty and TMD-P was 0.78, indicating a lower probability for TMD-P, the older a person is at puberty. Several other studies have found that prevalence increases with age for females, but not for males [9–11, 19]. Some studies suggest that pain in women with TMD may be enhanced during the low-estrogen phase of the menstrual cycle [29–31]. This finding indicates that puberty and hormones play a part in the onset of TMD-P, especially for females.

Other explanations for the differences in general are higher levels of stress and depression among females compared to males, although males report more frequent stress. Furthermore, females perceive pain as more severe and have a greater temporal summation of painful stimuli than males [12]. This finding is in accordance with the significantly higher pain intensity, measured by the VAS score among females compared to males in this study.

The overall VAS score indicated mild to moderate pain, but the range shows that some patients experienced high-intensity pain (Tables 2, 3, and 4).

A significant correlation was seen between frequent headaches and TMD-P. This correlation has been reported in several other studies [7, 9–11, 20, 32]. Nilsson et al. [32] found headaches to be the most important independent factor for developing TMD-P, and they reported that headache precedes TMD-P for most adolescents. The OPPERA study supports this as well, although it is not explicit TMD-P, especially for migraine type of headaches [33]. Coexistence of other bodily pain, especially neck, abdominal, and back pain, has been found to correlate with TMD [32, 34–38]. Myalgia was the most frequent symptom found in this study, which can possibly explain the correlation seen between headaches and TMD-P. The symptoms of headaches and TMD-P can be difficult to distinguish, and some of the TMD-P patients in this study may have had a headache condition and could be considered false positives. Tension-type headaches are the most common reason for false-positive results based on self-reported TMD-P questions [17]. The other Norwegian study using TMD-P found that four out of five patients with a positive TMD-P score had a diagnosis of a headache in the temporal region according to the DC/TMD protocol. Pain, such as a headache, gives central facilitation of nociceptive input from structures such as the muscles of mastication and seems to play a part, especially for muscle disorders [39]. This finding could indicate that one painful condition makes patients more susceptible to other painful conditions, which has been explained by differences in the endogenous pain-modulating system. Chronic long-standing pain decreases the endogenous system's ability to reduce pain sensation [40], which is in accordance with this study's finding of significantly more TMD-P in females reporting severe menstrual pain.

In contrast, physical activity could have an opposite effect on the endogenous pain-modulating system. Several studies have shown that regular exercise has a pain-reducing effect for patients with chronic pain [41, 42], which may explain the finding of significantly less TMD-P among patients in the current study who reported regular physical activity. Low physical activity has been found to correlate with chronic pain [40]. A study looking at the grade in physical education in high school adolescents found a correlation between low (bad) grades and the development of chronic painful musculoskeletal conditions in adulthood [42]. Our study did not find an association between spending more than 3 hours daily in front of a computer and TMD-P. According to the World Health Organization (WHO), individuals aged 5 to 17 years need 60 minutes of physical activity of moderate to vigorous intensity daily, while those who are 18 to 64 years old need 150 minutes a week with the same intensity [43]. Both age groups obtain additional health benefits from increasing physical training. The physical activity can be running, weightlifting, long fast walks, bicycling, or other things. WHO also recommends that adolescents have less than 2 hours of screen time daily, although it can be argued that adolescents spending more than 3 hours in front of a computer daily still have time to fulfill the WHO recommendations for physical activity. This argument is partly supported by a Spanish study that showed no significant association between increased screen time and low physical activity on weekend days. On weekdays, however, increased screen time seemed to displace time for physical activity [44]. The time limit of 3 hours set in our study was possibly too low to reveal any differences.

Two other important findings were the possible predisposing factors of living in urban areas and living in families with divorced or single parents. These findings indicate that environmental and cultural factors may play a role in the development and sustainment of TMD-P among adolescents. A larger American study (the OPPERA study) has reported similar findings [45], finding that the geographical study site was independently associated with TMD incidence. The same study found a relation between marital status and the onset of TMD, although for the participants themselves and not for the offspring, as in this study. One can still argue that some of the same possible mechanisms affect children, such as increased psychosocial stress due to loss of stability, changed housing, and lowered household income. Another part of the OPPERA study reported a higher incidence of TMD among individuals who reported dissatisfaction with the material standard of life [46]. However, the results from our study cannot be directly compared with the OPPERA study because we used different screening systems. A large Swedish study also found a difference in TMD-P for adolescents between urban and rural clinics [10]. A Finnish study on headache in urban and rural areas reported that headaches are significantly more common in urban than in rural areas [47]. Psychosocial factors seem to be a possible explanation for the difference in prevalence between urban and rural areas. According to a Norwegian national survey published in 2014, the proportion of adolescents with signs of depression increases from 9% in rural areas to 12% in urban areas [15]. It may be speculated that life in rural areas is more easy-going, with less emphasis on things like appearance and school performance.

Trauma to the face, and especially the jaw, has previously been proposed as a major risk factor for the development of TMD in adults [1]. In this study, none of the case patients with positive TMD-P reported trauma to the face, but 12 patients in the control group did. A possible explanation is that the participants in this study were young, and trauma as a cause or initiator of TMD-P takes many years to develop into a painful condition [1, 48].  Table 5 shows that symptoms like locking of the jaws and crepitation sounds from the joint were rare in the TMD-P group in this cohort. Internal derangement of the TMJ progresses slowly, and thus it is quite uncommon among young people [48, 49]. It cannot be excluded that patients in the control group will later develop TMD or TMD-P due to the previous trauma.

Strengths of this study are the large size of the study group and the use of a screening tool with high sensitivity and validity for TMD-P. Further strengths are the randomized and representative selection of patients, which means that the estimated prevalence of 7% should be a fair estimate of the actual prevalence of TMD-P among adolescents in Rogaland County, Norway.

A limitation of this study is the clinical examination having been performed by several dentists. Intraexaminer error cannot be excluded. Although we made an effort to calibrate how the dentists did the patient investigations, we did not perform any statistic evaluation of intraexaminer discrepancies. Another limitation is that we did not examine the control group with the DC/TMD protocol. That would have been useful for comparing this study to the other Norwegian study; however, our goal was to identify an easy screening tool for the dental clinics to find TMD-P at an early phase. TMD-P in adolescents needs treatment to prevent chronic pain in adulthood. There may have been patients with a TMD diagnosis based on the DC/TMD who might later seek treatment when the condition becomes painful. It is important to note that nonpainful TMD is not an issue for the patient and should therefore not be emphasized. There are studies showing that two-thirds of cases with chronic closed lock of the disc in TMJ will resolve over time, within an average of 6.12 months, without any treatment [50–52].

A further limitation is the age of the study group. These patients are still growing, and we cannot predict what will happen later. Undiagnosed conditions especially in the joints can have a negative impact on growth, and as seen in other studies, the prevalence of both TMD and TMD-P increases with age. Therefore, it is important to have easily used diagnostic tools such as TMD-P questions that can identify symptoms for early treatment. A follow-up study on the patients that have answered yes on TMD-P would be of great interest.

The TMD-P screening method can be useful for dentists in the PDS to aid in the diagnosis of painful TMD, especially when risk factors are present. This method can be a fast and cost-effective way to determine whether these patients should be examined more thoroughly and whether the use of the DC/TMD protocol is appropriate. A longitudinal follow-up of this study (cases and controls) would be interesting to determine whether painful TMD develops among the study patients in adulthood. So far, the longest follow-up study has been 2.8 years, with an incidence rate of 4% per annum [53].

## 5. Conclusions

The prevalence of TMD-P among adolescents found in this study was 7%. This prevalence is low, but it is comparable to studies in other countries using the same methodology. Being female, living in urban areas, having severe menstrual pain, and having frequent headaches and the parent marital status are all associated with TMD-P. Regular exercise is associated with less TMD-P. Females report significantly higher pain intensity than males. Myalgia is the most frequent symptom of TMD-P.

## Figures and Tables

**Table 1:** The frequency of different variables in the case group (TMD-P) versus control group.

Variable	Case group, %	Control group, %	*p* value (*χ*^2^)
General health factors			
Facial trauma	0	2.3	0.239
Whiplash injury	2.4	0.8	0.477
Frequent headache	43.9	19.3	<0.001^*∗*^
Severe menstrual pain (*n* = 286)	41.5	17.0	0.046^*∗*^
General joint hypermobility	2.0	1.0	0.597
General joint disease	2.8	1.5	0.173
Sinusitis	7.7	1.5	0.090
Ear infections	5.1	4.8	0.890
Throat infections	5.1	3.1	0.673
Allergy	33.3	34.7	0.859
Asthma	15.8	8.9	0.158
Previous orthodontic treatment	29.0	26.0	0.623
Lifestyle and social factors			
>3 h in front of computer per day	30.7	26.9	0.606
Divorced or single parents	51.3	30.1	0.008^*∗*^
Regular exercise	56	73.5	0.022^*∗*^

^*∗*^Statistically significant based on the Pearson chi-square test.

**Table 2:** Pain intensity scored on a VAS (0–100) for males and females analyzed with a *t*-test.

Sex	Mean	SD	95% CI	Max	Min	*p* value
Male	20.5	14.05	12.24–28.85	49	0	0.014^*∗*^
Female	35.4	19.68	28.25–42.58	75	1	

^*∗*^Statistically significant based on Student's *t*-test.

**Table 3:** Comparison of pain intensity using a VAS (0–100) for the diagnosis of myalgia, arthralgia, and a combination of both.

Diagnosis	Mean	SD	95% Ci	Max	Min	*p*value^*∗*^
Arthralgia	36.2	21.3	22.3–50.1	68	9	0.829
Combination	38.2	17.0	27.1–49.3	75	19	
Myalgia	26.9	19.2	18.7–35.2	68	0	0.129
Combination	38.2	17.0	27.1–49.3	75	19	
Myalgia	26.9	19.2	18.7–35.2	68	0	0.279
Arthralgia	36.2	21.3	22.3–50.1	68	9	

∗Based on Student's *t*-test.

**Table 4:** Comparison of pain intensity using a VAS (0–100) between males and females for the patients diagnosed with myalgia. There were insufficient data to compare males and females for arthralgia or a combination of muscle and joint pain.

	Mean	SD	95% CI	Max	Min	*p*value^*∗*^
Males	18.4	15.6	7.6–29.2	49	0	0.0923
Females	32.2	19.8	21.5–43.0	68	1	

^*∗*^Based on Student's *t*-test.

**Table 5:** Frequency of certain symptoms in the case group.

	Daily	Weekly	Seldom	Never
Headache	10%	35%	40%	15%
Locking	0%	17.5%	22.5%	60%
Tiredness of the face	7.5%	17.5%	25%	50%
Pain when opening wide and chewing	7.5%	45%	25%	22.5%
Difficulty opening wide	7.5%	10%	32.5%	50%
Clicking or popping	17.5%	22.5%	22.5%	37.5%
Crepitation	0%	5%	5%	90%
